# 16S rRNA amplicon sequencing reveals a polymicrobial nature of complicated claw horn disruption lesions and interdigital phlegmon in dairy cattle

**DOI:** 10.1038/s41598-018-33993-9

**Published:** 2018-10-19

**Authors:** V. Bay, B. Griffiths, S. Carter, N. J. Evans, L. Lenzi, R. C. Bicalho, G. Oikonomou

**Affiliations:** 1 Department of Epidemiology and Population Health, Institute of Infection and Global Health, University of Liverpool, Liverpool, UK; 2 Department of Livestock Health and Welfare, Institute of Veterinary Sciences, University of Liverpool, Liverpool, UK; 3 Department of Infection Biology, Institute of Infection and Global Health, University of Liverpool, Liverpool, UK; 4 Centre for Genomic Research, Institute of Integrative Biology, University of Liverpool, Liverpool, UK; 5 Department of Population Medicine and Diagnostic Sciences, College of Veterinary Medicine, Cornell University, Ithaca, USA

## Abstract

Lameness represents an intractable problem for the dairy industry. Complicated claw horn disruption lesions, interdigital hyperplasia, and interdigital phlegmon are important lameness causing foot lesions. Their aetiology is multifactorial, but infectious processes are likely implicated in disease pathogenesis. Our aim was to investigate the bacterial profiles of these lesions using 16S rRNA gene sequencing of samples obtained from 51 cattle across ten farms in the UK. In this study, interdigital hyperplasia, interdigital hyperplasia with signs of interdigital dermatitis, interdigital phlegmon, complicated sole ulcers, complicated toe ulcers lesions, and complicated white line lesions were investigated; corresponding healthy skin control samples were also analysed. All diseased tissues displayed reduced microbial richness and diversity (as described by Chao1, Shannon, and Simpson alpha-diversity indices) compared to their healthy skin control samples. Our results confirm the association of *Treponema* spp with some of these disorders. Other anaerobic bacteria including *Fusobacterium* spp., *Fastidiosipila* spp. and *Porphyromonas* spp. were implicated in the aetiology of all these lesions with the exception of interdigital hyperplasia. Complicated claw horn disruption lesions, and interdigital phlegmon were found to have similar bacterial profiles. Such sharing of bacterial genera suggests many of the infectious agents detected in these foot lesions are acting opportunistically; this finding could contribute towards future treatment and control strategies.

## Introduction

Lameness is a significant health issue within the dairy cattle industry due to reduced animal welfare and productivity, and associated economic losses^1,2^. Lameness, a clinical sign and not a disease per se, is multifactorial in nature with over 90% of lameness causing lesions found in the foot^3^. These lesions can have either an infectious or a non-infectious aetiology. The most common lesions of infectious aetiology are interdigital phlegmon (IP) (also known as foul in the foot, interdigital necrobacillosis or bovine footrot)^4^, and digital dermatitis (DD). The most common non-infectious lesions are described as claw horn disruption lesions (CHDL) and include sole hemorrhages (SH), sole ulcers (SU), toe ulcer/ necrosis (TU/ TN), and white line disease (WLD)^2,5,6^.

Bovine DD was reported for the first time in 1974^7^ and usually affects the palmar or plantar aspect of the feet, caudal to the interdigital space. *Treponema* spp. have been consistently detected in high numbers and routinely isolated from these lesions and are considered the primary aetiological agent^8–11^. Digital dermatitis associated *Treponema* spp. have also been associated with complicated (non-healing) CHDLs (complicated SU, TN and complicated WLD) as they have been shown capable of infecting exposed corium^12,13^. Evans *et al*.^12^ described a strong association between the presence of all three identified, cultivable, DD treponemes; *Treponema medium-like*, *Treponema phagedenis-like* and *Treponema denticola-like* spirochaetes, within each of the three different non-healing bovine claw horn lesions: TN; non-healing WLD, and non-healing SU. In contrast to the typical CHDL lesions (which are of non-infectious aetiology), complicated lesions may display a topical granular appearance, with a typical pungent smell; presence of purulent discharge is also common^12,14^.

The infectious foot lesion IP is an acute or subacute necrotizing dermatitis located in the interdigital space. This lesion is considered to be caused by *Fusobacterium necrophorum*; however, *Porphyromonas levii* and *Prevotella intermedia* bacteria have also been isolated from these lesions^15–18^. Interdigital hyperplasia (IH) refers to the formation of hyperplastic interdigital skin at the axial hoof wall in the interdigital space^19^. Despite being a prevalent lesion, its aetiopathogenesis is not fully elucidated. It has been speculated that outwards spreading of the claws and poor ligamentous structure leads to stretching of the interdigital skin resulting in hyperplasia^20^. A potential association between IH and DD has been speculated; however, evidence supporting this association remains scarce^4,21,22^.

Culture-independent analysis of mixed microbial communities relying either on target-specific 16S rRNA gene sequencing or shotgun metagenomic sequencing^23,24^ can aid the study of diseases of a potentially polymicrobial nature and lead to a better understanding of their aetiopathogenesis. *Treponema* spp. have recently been associated with bovine DD lesions using 16S rRNA gene metagenomic sequencing^25–27^. However, studies of the microbial communities of complicated CHDL, IP or IH lesions are still absent from the scientific literature. It could be valuable to characterise the bacterial composition of these lesions in order to identify microorganisms involved in disease pathogenesis.

We performed amplicon-based 16S rRNA gene sequencing of DNA extracted from swab samples collected from dairy cows affected with IH, infected IH, IP, and complicated SU, TN, and WLD lesions. DNA was also collected from healthy skin control swabs and was also analysed for comparative purposes. Our objective was to describe the bacterial composition of each lesion type, and to identify the putative pathogens involved.

## Results

Swab samples were collected from 10 dairy farms in Cheshire and North Wales, UK. The numbers of each lameness causing lesion obtained from each different farm are described in Table 1. All collected samples were eventually submitted for sequencing.

**Table 1:** Distribution of lesions in each farm.

Farm	IH	IHC	IIH	IIHC	IP	IPC	SU	SUC	TN	TNC	WLD	WLDC
A	0	0	0	0	0	0	1	1	0	0	0	0
B	0	0	0	0	3	2	0	0	0	0	4	4
C	0	0	0	0	0	0	1	0	0	0	1	1
D	6	6	2	2	0	0	0	0	0	0	0	0
E	5	5	2	2	0	0	0	0	0	0	0	0
F	0	0	0	0	0	0	4	4	0	0	3	3
G	0	0	0	0	0	0	0	0	0	0	2	2
H	0	0	0	0	0	0	0	0	0	0	1	1
I	0	0	0	0	0	0	0	0	2	2	0	0
J	0	0	0	0	0	0	14	14	1	1	0	0
Total	11	11	4	4	3	2	20	19	3	3	11	11

(IH: Interdigital Hyperplasia, IHC: IH Control IIH: Infected Interdigital Hyperplasia, IIHC: IIH Control, IP: Interdigital Phlegmon, IPC: IP Control, SU: Sole Ulcer, SUC: SU Control, TN: Toe Necrosis, TNC: TN Control, WLD: White Line Disease, WLDC: WLD Control).

**Figure 1:**
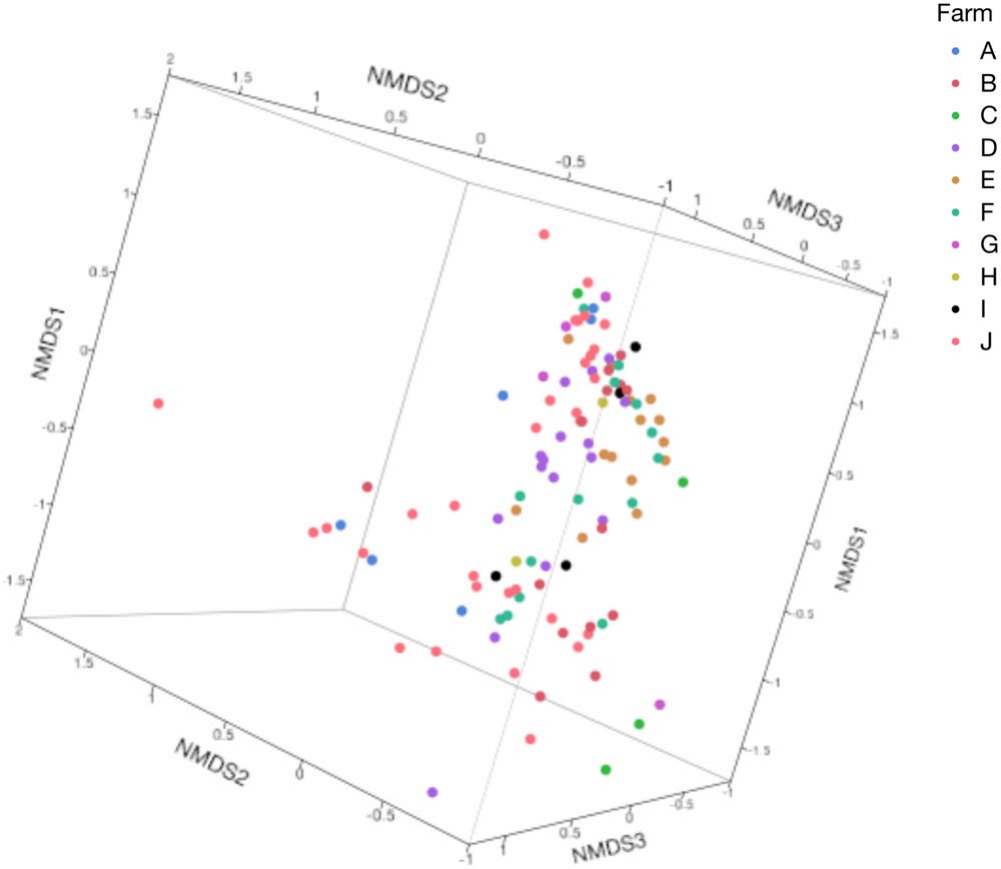
NMDS plot of weighted UniFrac distances for samples obtained from different farms (Analysis of Similarity (ANOSIM), R = 0.33, P = 0.001).

**Figure 2:**
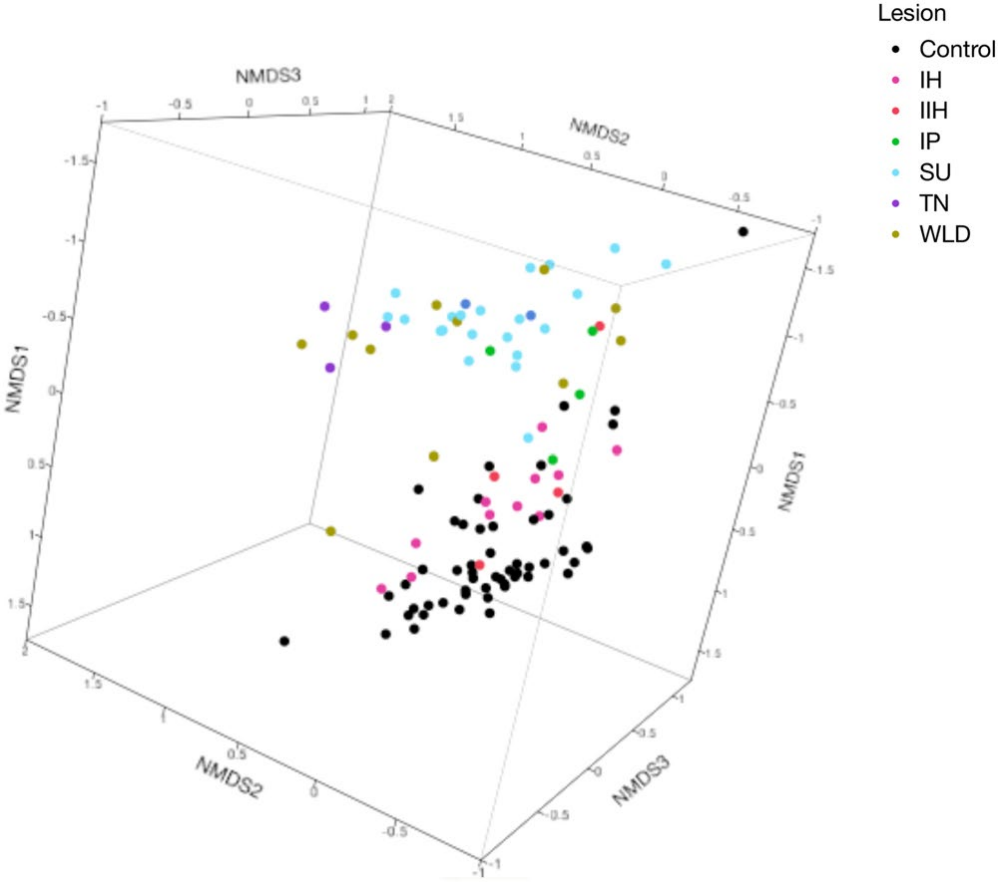
NMDS plot of weighted UniFrac distances of all lesions and their all healthy skin control samples (Analysis of Similarity (ANOSIM) R = 0.61, P = 0.001). (IH: Interdigital Hyperplasia, IIH: Infected Interdigital Hyperplasia, IP: Interdigital Phlegmon, SU: Sole Ulcer, TN: Toe Necrosis, WLD: White Line Disease).

After quality filtering processes, a total of 47,661,917 sequences were used for further analyses (Mean = 433,290, SD = 58,425 sequences per sample). Chao1, Shannon, and Simpson alpha-diversity indices for different lesion groups and their control samples are shown in Table 2. Beta-diversity was calculated through weighted UniFrac distances, and non-metric multidimensional scaling (NMDS) values were charted in 3D scatterplots. Bacterial composition of the samples did not indicate any clear clustering between farms (Fig. 1). On the other hand, there was a clear distinction between healthy skin control samples and IP, SU, TN, and WLD lesions. IH and IIH samples were however grouped together with the control samples (Fig. 2).

**Table 2:** Alpha diversity analyses of all lesions compared to their healthy skin control samples.

	Chao1	*P*-value	Shannon	*P*-value	Simpson	*P*-value
IH	16496.14 ± 2952.34	1.00	9.72 ± 0.47	0.10	**0**.**02** ± **0**.**01**	**0**.**04**
IHC	16593.19 ± 2982.26		10.26 ± 0.65		**0**.**03** ± **0**.**02**	
IIH	16791.32 ± 2013.63	0.99	9.56 ± 1.16	0.35	0.02 ± 0.01	0.75
IIHC	16593.19 ± 2982.26		10.26 ± 0.65		0.02 ± 0.01	
IP	9695.06 ± 2326.01	0.14	7.08 ± 1.72	0.28	0.01 ± 0.00	0.15
IPC	18163.51 ± 404.56		10.76 ± 0.05		0.03 ± 0.00	
SU	11036.77 ± 4006.81	0.09	**7**.**54** ± **1**.**11**	**0**.**01**	**0**.**01** ± **0**.**00**	**0**.**02**
SUC	18318.57 ± 2461.62		**10**.**34** ± **0**.**60**		**0**.**02** ± **0**.**01**	
TN	7812.94 ± 1988.10	0.09	6.62 ± 0.55	0.09	0.01 ± 0.00	0.06
TNC	18446.32 ± 720.82		10.51 ± 0.37		0.04 ± 0.01	
**WLD**	**8930**.**12** ± **1956**.**27**	**0**.**01**	**6**.**97** ± **0**.**68**	**0**.**02**	**0**.**01** ± **0**.**00**	**0**.**03**
**WLDC**	**17842**.**96** ± **1489**.**88**		**10**.**51** ± **0**.**47**		**0**.**03** ± **0**.**01**	

Comparisons were made with the use of a series of t-tests. (IH: Interdigital Hyperplasia, IHC: IH Control IIH: Infected Interdigital Hyperplasia, IIHC: IIH Control, IP: Interdigital Phlegmon, IPC: IP Control, SU: Sole Ulcer, SUC: SU Control, TN: Toe Necrosis, TNC: TN Control, WLD: White Line Disease, WLDC: WLD Control). Statistically significant comparisons (*P* < 0.05) are highlighted in bold.

**Figure 3:**
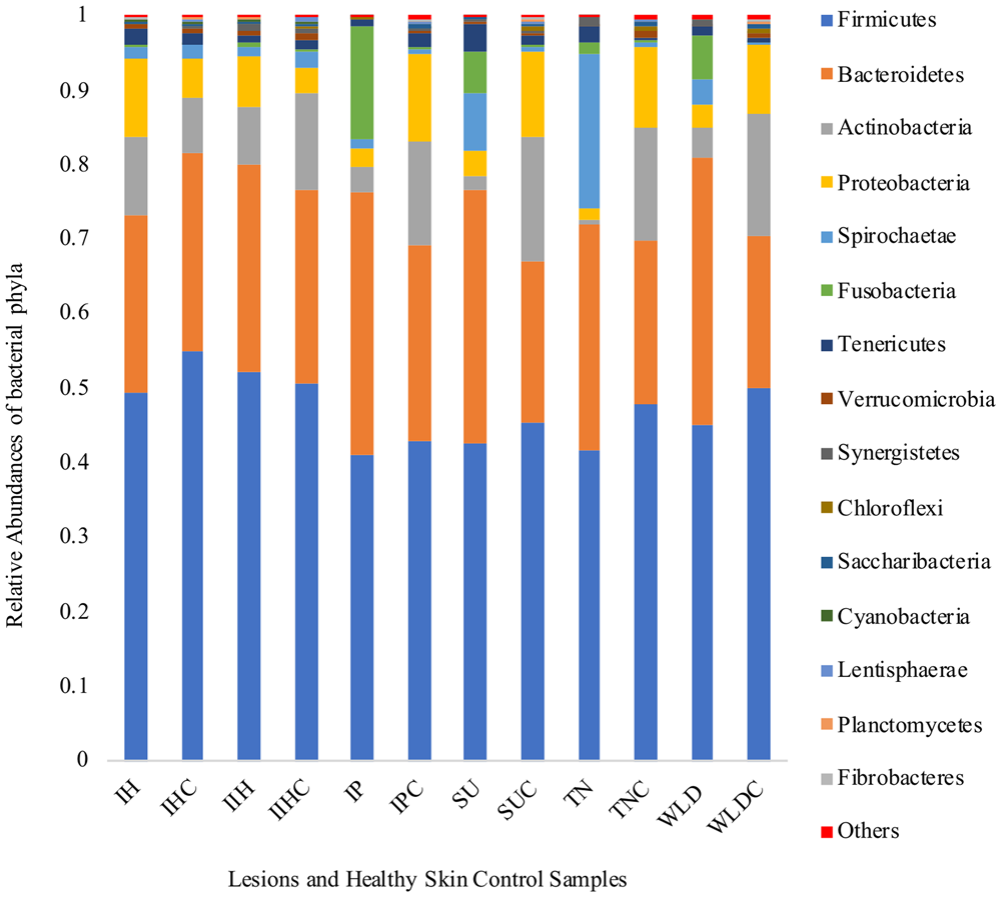
Relative abundances of fifteen most prevalent bacterial phyla in lesion and healthy skin control samples. (IH: Interdigital Hyperplasia, IHC: IH Control IIH: Infected Interdigital Hyperplasia, IIHC: IIH Control, IP: Interdigital Phlegmon, IPC: IP Control, SU: Sole Ulcer, SUC: SU Control, TN: Toe Necrosis, TNC: TN Control, WLD: White Line Disease, WLDC: WLD Control).

**Figure 4:**
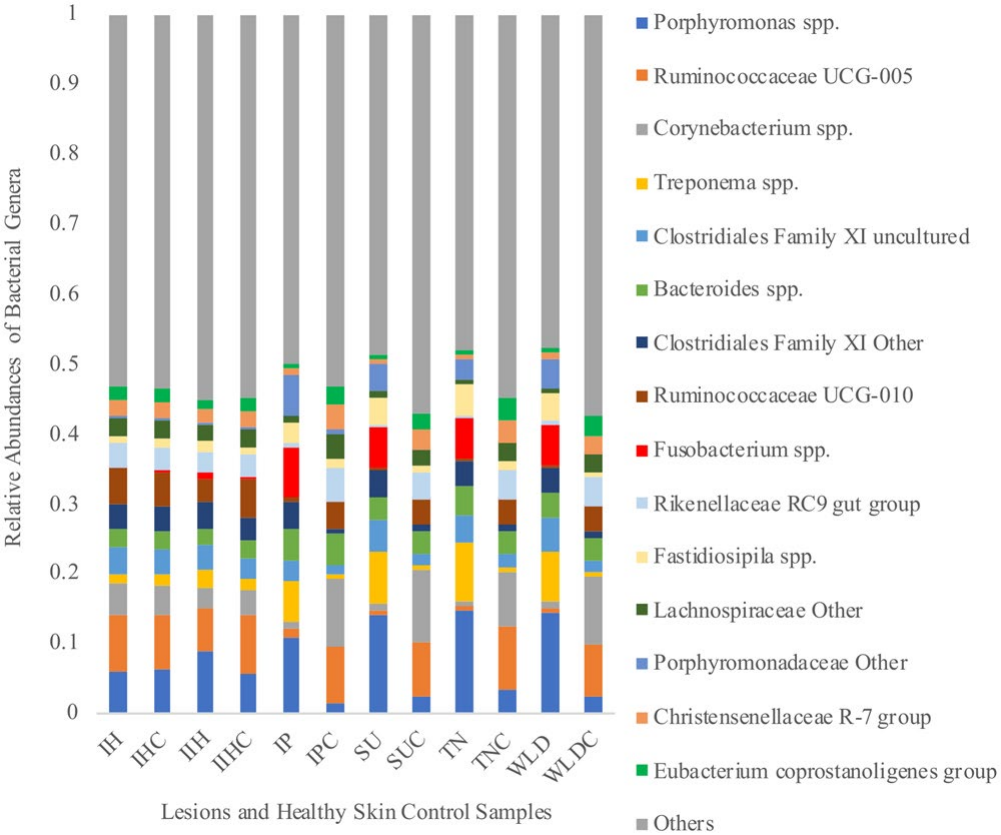
Relative abundances of fifteen most prevalent bacterial genera in lesion and healthy skin control samples. (IH: Interdigital Hyperplasia, IHC: IH Control IIH: Infected Interdigital Hyperplasia, IIHC: IIH Control, IP: Interdigital Phlegmon, IPC: IP Control, SU: Sole Ulcer, SUC: SU Control, TN: Toe Necrosis, TNC: TN Control, WLD: White Line Disease, WLDC: WLD Control).

Relative abundances of the 15 most prevalent phyla are shown in Fig. 3. Main phyla for all the samples were Firmicutes followed by Bacteroidetes. Spirochaetes and Fusobacteria phyla were found in increased relative abundances in SU, TN, and WLD lesions compared to their control samples. In Fig. 4, relative abundances of the 15 most prevalent genera are shown.

**Figure 5:**
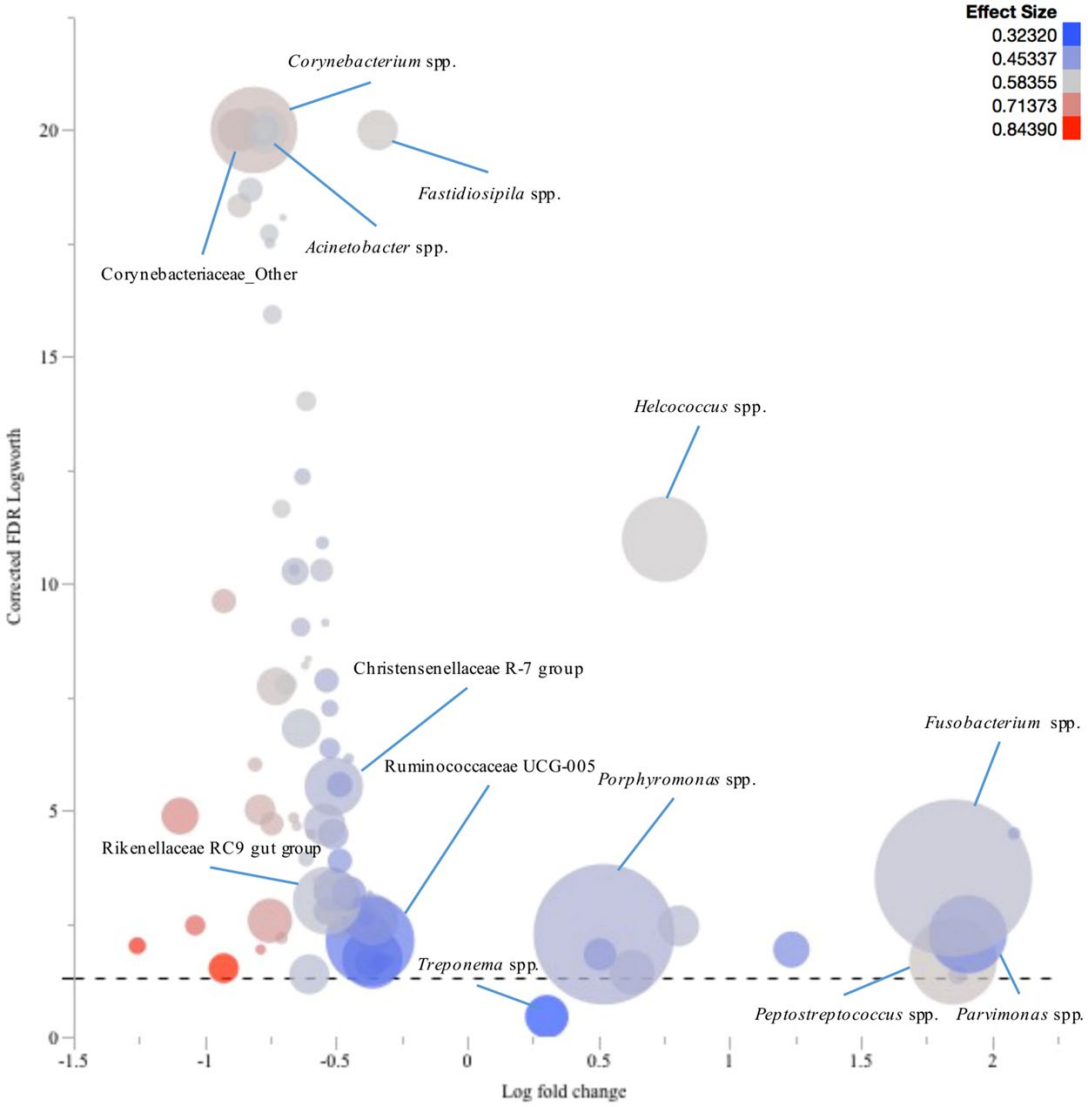
Comparison of the microbiota profiles of interdigital phlegmon (IP) samples and their healthy skin control samples. The log fold change in genera relative abundances in samples from IP lesions comparing to their healthy skin control samples is plotted versus the corrected robust false discovery rate (FDR) logWorth (i.e. log10P). The dashed line shows the P-values (0.05) adjusted for FDR. Size of the circles represents the mean relative abundance of each genus, and colour represents the effect size.

The response screening analysis results provided a more comprehensive analysis of the differences at genus level between lesion and control samples; these results are charted in bubble plots. *Fusobacterium* spp., *Porphyromonas* spp., *Helcococcus* spp., *Parvimonas* spp., and *Peptostreptococcus* spp. were found to be significantly more prevalent in IP lesion samples compared to their healthy skin control samples. On the other hand, the prevalence of *Corynebacterium* spp., *Acinetobacter* spp., Christensenellaceae R-7 group, Ruminococcaceae UCG-005, Rikenellaceae RC9 gut group, and other genera of the family Corynebacteriaceae were significantly higher in healthy skin control samples compared to IP lesion samples (Fig. 5).

**Figure 6:**
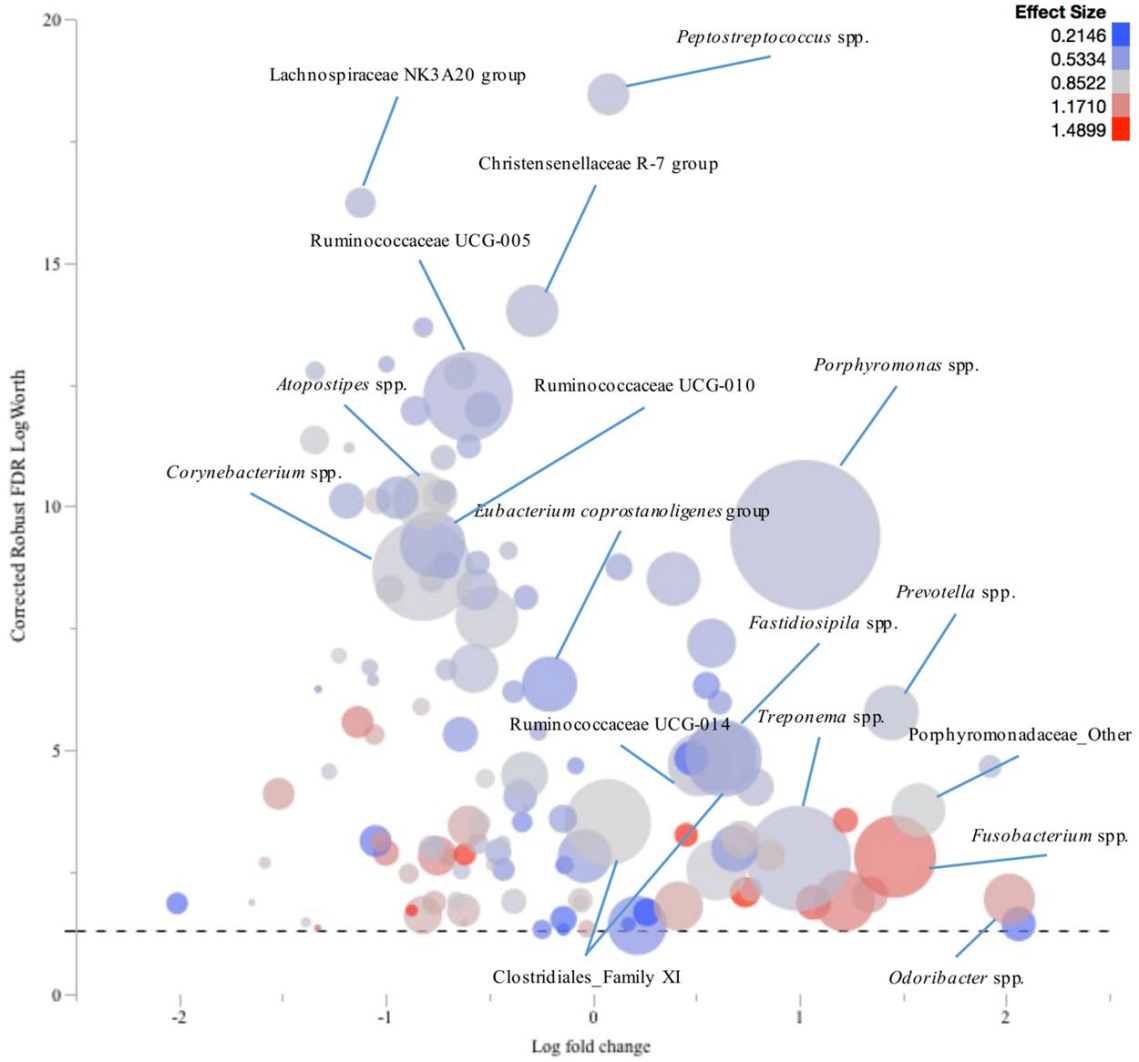
Comparison of the microbiota profiles of sole ulcer (SU) samples and their healthy skin control samples. The log fold change in genera relative abundances in samples from SU lesions comparing to their healthy skin control samples is plotted versus the corrected robust false discovery rate (FDR) logWorth (i.e. log10P). The dashed line shows the P-values (0.05) adjusted for FDR. Size of the circles represents the mean relative abundance of each genus, and colour represents the effect size.

In SU lesion samples, *Fusobacterium* spp., *Porphyromonas* spp., *Treponema* spp., *Murdochiella* spp., *Odoribacter* spp., *Peptostreptococcus* spp., *Ezakiella* spp., *Prevotella* spp., and Clostridiales Family XI were significantly more abundant compared to their healthy skin control samples. The prevalence of *Corynebacterium* spp., Ruminococcaceae UCG-005, Christensenellaceae R-7 group, *Eubacterium coprostanoligenes*, and Lachnospiraceae NK3A20 group was significantly higher in the healthy skin samples compared to the SU lesion ones (Fig. 6).

**Figure 7:**
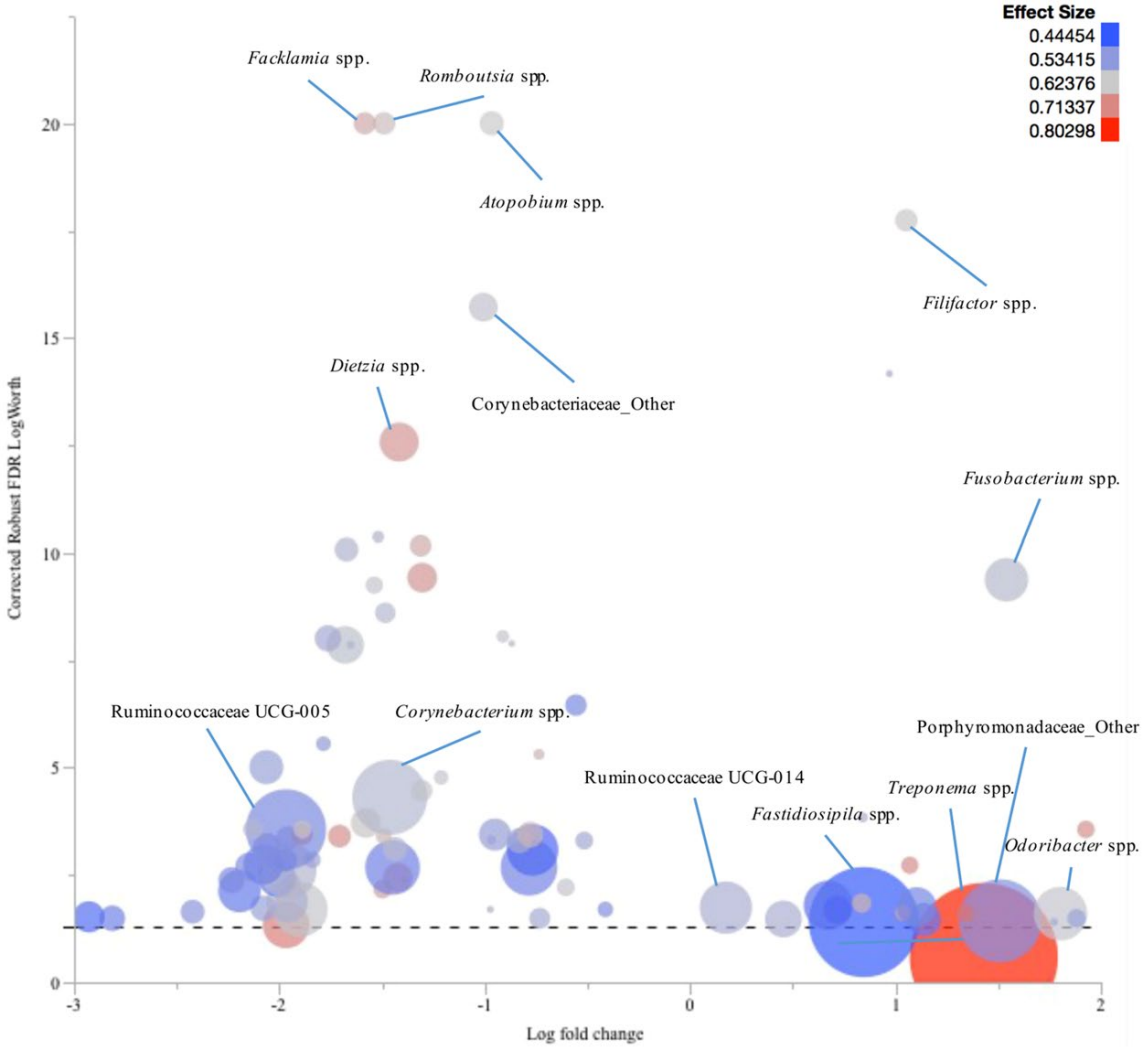
Comparison of the microbiota profiles of toe necrosis (TN) samples and their healthy skin control samples. The log fold change in genera relative abundances in samples from TN lesions comparing to their healthy skin control samples is plotted versus the corrected robust false discovery rate (FDR) logWorth (i.e. log10P). The dashed line shows the P-values (0.05) adjusted for FDR. Size of the circles represents the mean relative abundance of each genus, and colour represents the effect size.

In TN lesion samples, *Fusobacterium* spp., *Treponema* spp., *Fastidiosipila* spp., *Odoribacter* spp., *Filifactor* spp., Ruminococcaceae UCG-014 and other genera of the family Porphyromonadaceae were significantly more prevalent compared to their healthy skin control samples. *Corynebacterium* spp., *Dietzia* spp., *Facklamia* spp., *Romboutsia* spp., *Atopobium* spp., Ruminococcaceae UCG-005 and other genera of the family Corynebacteriaceae were more prevalent in the respective healthy skin control samples compared to TN lesion samples (Fig. 7).

**Figure 8:**
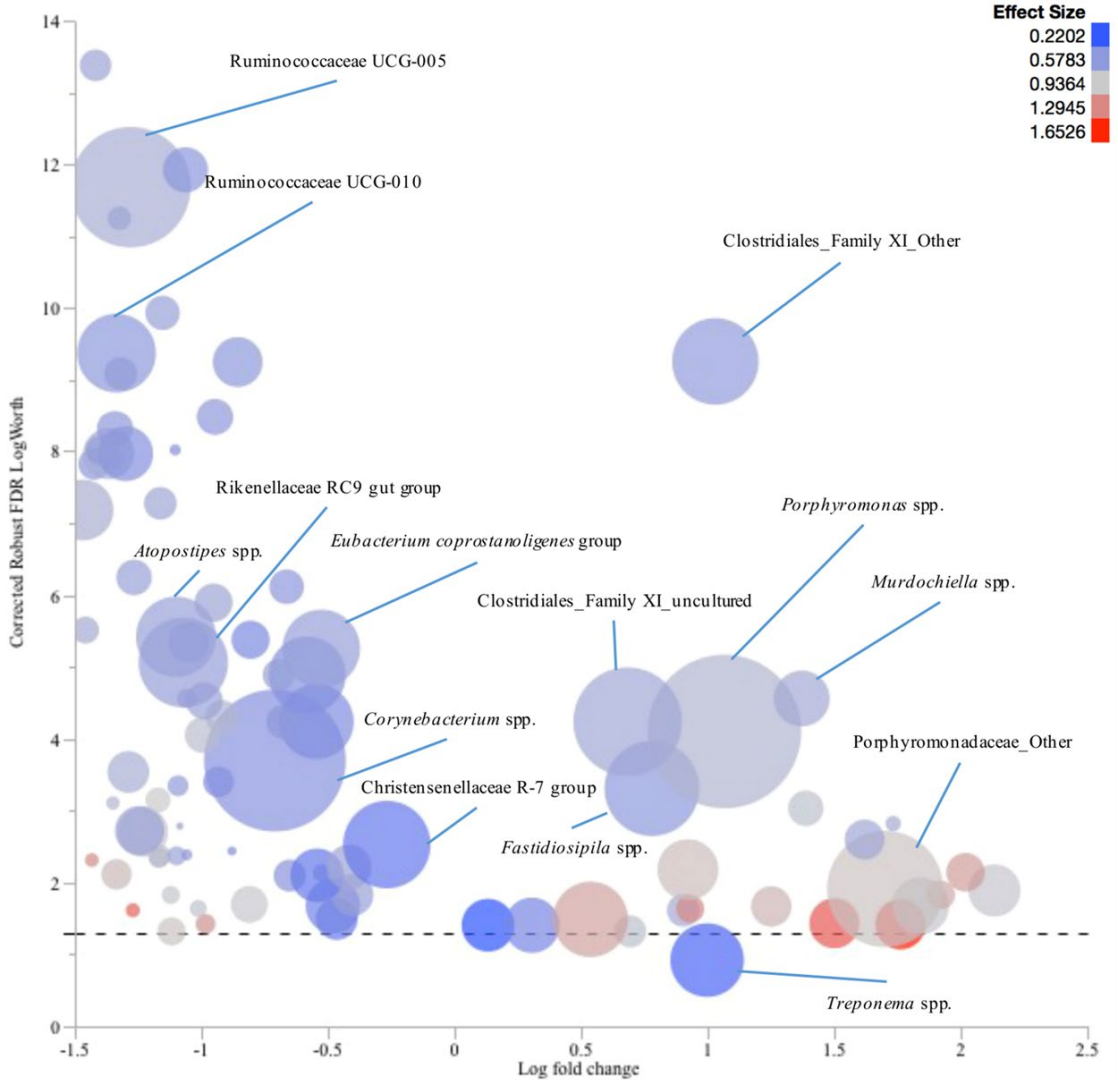
Comparison of the microbiota profiles of complicated white line disease (WLD) samples and their healthy skin control samples. The log fold change in genera relative abundances in samples from WLD lesions comparing to their healthy skin control samples is plotted versus the corrected robust false discovery rate (FDR) logWorth (i.e. log10P). The dashed line shows the P-values (0.05) adjusted for FDR. Size of the circles represents the mean relative abundance of each genus, and colour represents the effect size.

In WLD lesion samples, *Porphyromonas* spp., *Murdochiella* spp., *Fastidiosipila* spp., Clostridiales Family XI, and other genera of the family Porphyromonadaceae, were significantly more prevalent compared to their healthy skin control samples, while the prevalence of *Corynebacterium* spp., *Atopostipes* spp., Ruminococcaceae UCG-005, Ruminococcaceae UCG-010, Christensenellaceae R-7 group, *Eubacterium coprostanoligenes* group, and Rikenellaceae RC9 gut group were higher in healthy skin control samples compared to WLD lesion samples (Fig. 8).

**Figure 9:**
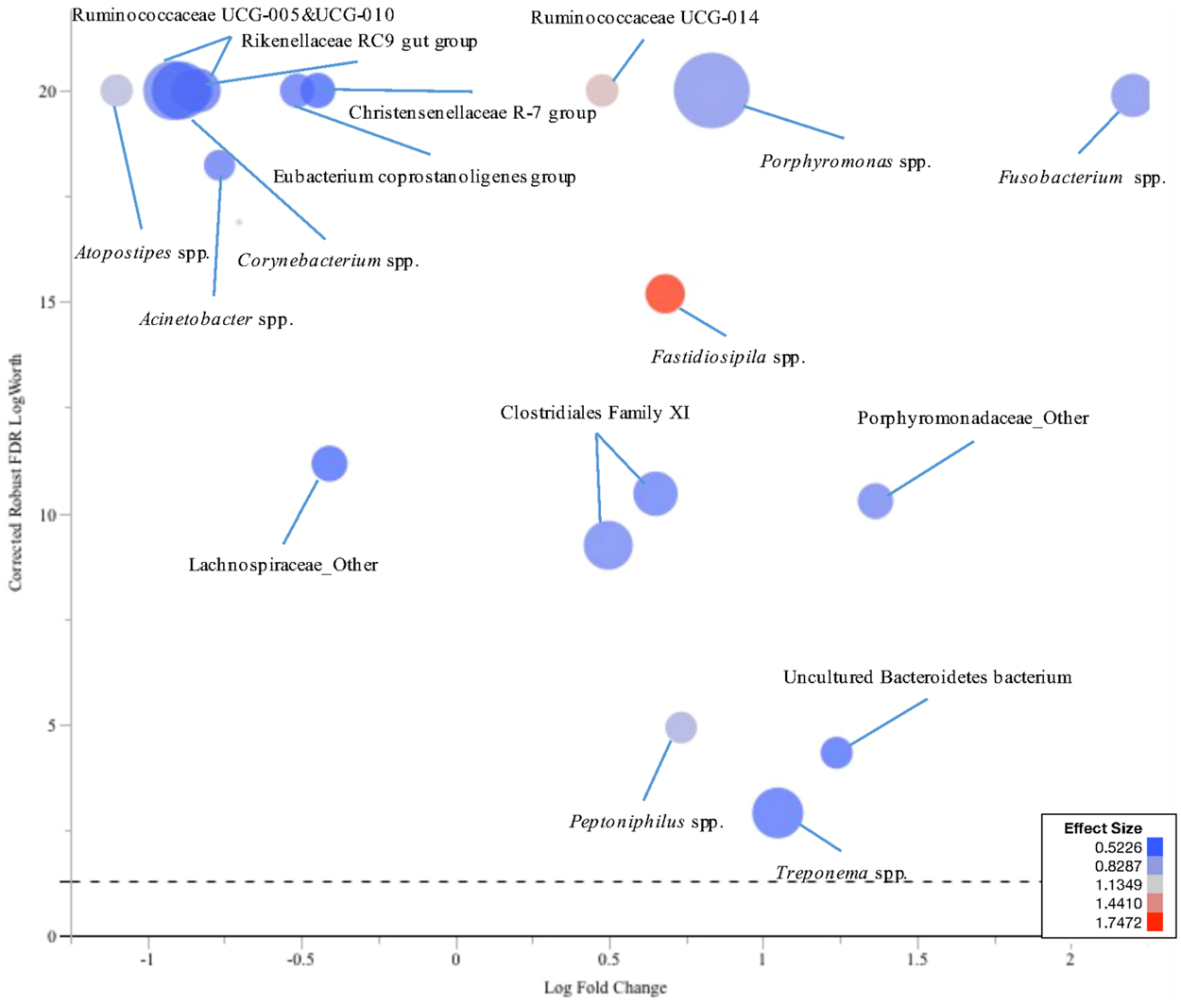
Comparison of the microbiota profiles of IP, SU, TN, and WLD lesion samples and their healthy skin control samples. Size of the circle represents the prevalence of each genera, and colour represents the effect size. The graph shows log fold change in 16S rRNA gene abundance in IP, SU, TN, and WLD lesions relative to their healthy skin control samples versus corrected robust false discovery rate (FDR) logWorth (i.e. log10P). The dashed line shows the P-values (0.05) adjusted for FDR.

Analysis of weighted UniFrac distances suggested that the microbial communities of complicated CHDL and IP lesions were similar. For this reason, response screening analysis was also performed for all the complicated CHDL and IP lesions together comparing them to all their healthy skin control samples. Several genera showed significant differences between lesions and control samples, therefore only the genera with mean relative abundance higher than 0.01 were charted in order to decrease complexity. *Porphyromonas* spp., *Fusobacterium* spp., *Treponema* spp., Clostridiales Family XI, *Fastidiosipila* spp., *Peptoniphilus* spp., Ruminococcaceae UCG-014, uncultured Bacteroidetes bacterium, and other genera of the family Porphyromonadaceae were found to be significantly more prevalent in lesion samples compared to healthy skin control samples. On the other hand, the prevalence of *Corynebacterium* spp., *Atopostipes* spp., *Acinetobacter* spp., Ruminococcaceae UCG-005, Ruminococcaceae UCG-010, Christensenellaceae R-7 group, *Eubacterium coprostanoligenes* group, Rikenellaceae RC9 gut group, and other genera of the family Lachnospiraceae were significantly higher in healthy skin control samples compared to lesion samples (Fig. 9).

In IH lesions, *Erysipelothrix* spp., *Guggenheimella* spp., *Peptococcus* spp., *Petrimonas* spp., Clostridiales Family XI, and other genera of the family Porphyromonadaceae were significantly more prevalent compared to their healthy skin control samples. However, their relative abundance was found to be low. The prevalence of *Alistipes* spp., *Bacteroides* spp., Christensenellaceae R-7 group, *Eubacterium coprostanoligenes*, and Clostridiales Family XIII AD3011 group were significantly higher in healthy skin control samples compared to IH lesion samples. Lastly, *Peptoniphilus* spp. were significantly more prevalent in IIH lesions than their healthy control samples.

Besides the lesion and healthy skin control samples, the ZymoBIOMICS™ Microbial Community DNA Standard was used as a mock microbial community. This community comprises eight microbial and two fungal strains; seven out of eight bacterial strains were successfully assigned at species level, and in their expected relative abundances. One bacterium was correctly identified at the genus level. Only 0.05% of the sequences from the positive control sample were assigned to other unexpected bacteria. Despite being in extremely low concentrations, and not visible on the agarose gel, three PCR negative controls were also sequenced. Two of these negative controls yielded less than 1,000 sequences, and one of them yielded 20,951 sequences; less than 5% of the average number of sequences obtained from the lesion and healthy skin samples. In addition to these, sterile swabs were processed as negative DNA extraction controls. Since their concentrations were very low and they were invisible in the gel, these samples were not further analysed.

## Discussion

We have investigated the bacterial composition of complicated CHDL, IP, and IH samples and compared them to their healthy skin control samples. 16S rRNA gene sequencing revealed that all lesions are of polymicrobial nature rather than being associated with single taxa. Bacterial profiles of the healthy skin control samples were significantly different from lesion samples (with the exception of IH and IIH samples). In addition, healthy skin samples displayed an increased diversity compared to samples obtained from lesions. Recent studies on bovine mastitis have shown similar findings regarding microbial diversity of diseased versus healthy control samples^28^. As shown in previous studies, *Treponema* spp. appear to play an important role in the aetiology of some of these lesions^12,29^; however, other anaerobic bacteria such as *Fusobacterium necrophorum*, *Fastidiosipila* spp. and *Porphyromonas* spp. were also found to be highly prevalent in most of the studied lesions. Complicated CHDL were previously shown to be associated with DD *Treponema* spp. using species-specific PCR primers^12^. Here, we have used universal primers that allowed us to detect other bacteria that could potentially be associated with the progression of these lesions. Our results indicated that *Treponema* spp. were statistically significantly more prevalent in complicated SU lesions comparing to their corresponding healthy skin control samples. This was not the case for complicated WLD lesions. *Treponema* spp. were prevalent only in a few of the WLD lesions; their relative abundance across all the WLD samples was not statistically significantly different to their corresponding healthy skin control samples. Results associated with IIH, IP and TN should be treated cautiously since we only managed to obtain a small number of samples from these lesions.

The cross sectional design of our study only allows us to describe a snapshot of the differences in microbiota profiles and therefore we cannot make assumptions regarding the importance of different taxa at different time points of disease progression. In some of our cases, a specific pathogen (e.g. *Fusobacterium* spp. in IP) could have been primarily responsible for the lesion which was then also colonized by other opportunistic pathogens. CHDLs are considered to be of non – infectious aetiology and what we describe here is most likely the secondary invasion of the exposed corium by a number of different opportunistic bacteria. Further, larger scale, longitudinal studies could better elucidate these diseases’ aetiopathogenesis. A shotgun metagenomics approach could also be employed and would allow for a more in-depth investigation of the studied lesions’ microbial communities.

Spirochetes of the genus *Treponema* were previously described as the predominant bacteria in lameness associated DD lesions^30–32^. In this study, they were found to have significantly higher relative abundance in SU and TN samples compared to their healthy skin control samples. *T*. *medium*, *Treponema* phylotype 18, and other *Treponema* spp. were significantly prevalent in SU and TN lesions. *Treponema* phylotype 18 was shown to be sharing 95% sequence identity with recognized *Treponema* species (*T*. *putidum* ATCC 700334, *T*. *pedis* T3552B, and *T*. *denticola* ATCC 35405)^33^. Toe necrosis lesions were also populated by *T*. *denticola*, and *Treponema* canine oral taxon 233. The detrimental effects of *T*. *medium*, and *T*. *denticola* were previously described in human oral lesions^34,35^, bovine digital dermatitis^9^, and contagious ovine digital dermatitis (CODD)^36^. However, it should be noted that *Treponema* spp. were only significantly prevalent in a small number of the TN and WLD samples within this study; in many of them we were not able to detect *Treponema* spp. sequences (Supplementary Figures 3, 4) and this would suggest that they are acting opportunistically in these cases that can also be complicated by other pathogens.

*Porphyromonas* spp. were formerly isolated from different human and animal infections^37,38^. In our study, all lesions were mainly dominated by *P*. *levii* which has the ability to synthesize anti-IgG_2_ protease, and reduce macrophage activity^39^. *P*. *levii* is a well known opportunistic pathogen and is also commonly found in the vaginal discharge of metritic cows^40^. Complicated SU, TN, and WLD samples were also harbouring *Odoribacter denticanis*, which belongs to the same family (Porphyromonoadaceae) and was also previously associated with DD^41^. *Fusobacterium* spp., another well known opportunistic anaerobic pathogen, were previously found to be associated with lameness, particularly with DD and IP lesions^42,43^. Our results confirm its potentially important role in the development of IP but also indicated the significant presence of *Fusobacterium necrophorum* in all the other studied lesions. Invasion and colonization of tissues by *Fusobacterium necrophorum* is mediated by its virulence factors such as endotoxin, leukotoxin, and secreted proteases^44–46^. Its role in the aetiopathogenesis of dairy cattle metritis is also well known^47,48^.

Several types of bacteria in the Clostridiales order of the Firmicutes phylum were shown to have significant prevalence in both lesions and healthy skin control samples. One of these; *Fastidiosipila* spp. was previously reported in human osteitis^49^, and was found here in higher relative abundance in SU, TN, and WLD lesions (compared to healthy skin samples). Therefore, *Fastidiosipila* spp. could have a potentially important role in the aetiology of these lesions. Moreover, Clostridiales Family XI was significantly more abundant in SU, and WLD lesions (comparing to respective control samples); *Helcococcus* spp., and *Parvimonas* spp. from the same family were significantly more abundant in IP lesions. *Murdochiella* spp. were significantly more prevalent in WLD lesions and also belong to the Clostridiales family which is known to be associated with many human and animal diseases^50,51^.

Complicated CHDL and IP samples displayed similar microbiota profiles. Therefore, all these lesions were analysed together and compared to all control samples. Similarly to individual analysis of these lesions, the prevalence of *Fusobacterium necrophorum*, *Porphyromonas spp*., *Fastidiosipila* spp., and *Treponema* spp. was significantly higher in lesion samples compared to the prevalence of these bacteria in healthy skin control samples.

Conversely, our weighted UniFrac analysis suggested that IH and IIH samples were grouped together with healthy skin control samples. Therefore, these lesions might not be a result of bacterial infection. Alternatively, the causative bacteria in these lesions might be mainly located deep inside the lesions, and thus biopsy samples would have been more appropriate in order to identify them. Viral infections could also be implicated. *Erysipelothrix* spp. observed in these lesions, are known to cause erysipelas in pigs and are associated with acute septicaemia, cutaneous lesions, abortions, or chronic infection causing endocarditis and arthritis^52^. Moreover, *Guggenheimella* spp. were previously reported in DD lesions^53^, but for other lesions in this study (IP, TN, WLD samples), *Guggenheimella* spp. were observed in higher relative abundances in healthy skin control samples. A notable presence of *Porphyromonas* spp., and *Treponema* spp. was also observed in IH and IIH samples, but the difference between lesions and healthy skin control samples was not statistically significant.

In conclusion, we have characterised the bacterial profiles of six different lameness causing lesions using 16S rRNA gene amplicon sequencing and have used multivariate analysis approaches to analyse our data and described significant differences between lesions and their control samples. Our results showed that most of these lesions were associated with a range of pathogens, which are most likely acting opportunistically. *Porphyromonas* spp. were more prevalent in IP, SU, and WLD lesions, *Fusobacterium* spp. were more prevalent in IP, and SU lesions, and *Treponema* spp. were more prevalent in SU samples (compared to their respective healthy skin control samples). *Fastidiosipila* spp., a pathogen not previously associated with lameness causing lesions in cattle, showed a noteworthy prevalence in SU, TN, and WLD lesions. Our findings could contribute towards future treatment and control strategies for these disorders.

## Methods

### Ethics and sampling

Ethical approval for the study was granted by the University of Liverpool Research Ethics Committee (Reference Number: VREC547) all methods were performed in accordance with the relevant guidelines and regulations. As part of this cross- sectional study, a member of the research team accompanied five different professional foot trimmers as they visited clients’ farms throughout June and July 2017. Farm visits were organised to coincide with therapeutic foot trimming, as opposed to routine preventative foot trimming visits, to increase success of finding targeted lesions. Lesions targeted within this study included; complicated SU, complicated WLD, TN, IH, IH with signs of interdigital dermatitis, and IP. Cases were defined following the ICAR Claw Health Atlas definitions^54^ with the only additional requirement for a CHDL to be enrolled being the presense of obvious signs of infection. Claw horn disruption lesions without signs of infection (not complicated) were not included in this study. The foot trimmer was observed during routine foot trimming and when a targeted lesion was identified, a picture was taken, and a sterile swab used to obtain a sample from within lesion. In the case of IP and IH the skin was cleaned from any gross contamination with the use of a paper towel before sample collection. A second swab sample was taken from the plantar/palmar aspect of the affected foot, targeting the normal skin proximal and adjacent to the interdigital cleft, just above the heel bulbs. The area was cleaned from any gross contamination with the use of a paper towel before sample collection. Once obtained, swabs were placed in sterile tubes and labelled with lesion type, animal id, date, and whether the swab was lesion or control sample. Samples were transported on ice and stored at −80 °C until DNA extraction.

### DNA extraction

Microbial DNA was extracted from collected swabs using the PureLink™ Microbiome DNA Kit (Invitrogen, Carlsbad, CA, USA) and following the manufacturer’s instructions. The kit provides both enzymatical and mechanical disruption with bead beating. DNA extraction was performed under a laminar flow hood. Extracted DNA samples were stored at −20 °C.

### 16S rRNA gene amplification, and library construction

The Qubit™ dsDNA HS Assay Kit (Thermo Fisher Scientific, Fair Lawn, NJ, USA) was used to measure DNA concentrations prior to PCR amplification. The sample with the lowest DNA concentration (3.2 ng/μl) was used in maximum volume (10 μl) for PCR reaction (to equalize the amount of DNA in each tube) 32 ng from each sample was used as template DNA for amplification of the V3-V4 hypervariable region of 16S rRNA gene. Negative (Nuclease-Free Water (not DEPC-Treated) Thermo) and positive controls (ZymoBIOMICS™ Microbial Community DNA Standard) were also amplified. The 341 F and 805 R universal primers were used^55^. For the first step PCR, 1.25 μl of amplicon PCR forward primer (2.5 μM), 1.25 μl of amplicon PCR reverse primer (2.5 μM), and 12.5 μl of NEBNext High-Fidelity 2X PCR Master Mix (New England Biolabs) were used at 95 °C initial denaturation for 3 min, followed by 12 cycles of 95 °C for 30 s, 62.3 °C for 30 s, and 72 °C for 30 s, and a final extension at 72 °C for 5 min^55^. PCR products were cleaned up with Agencourt AMPure XP beads (Beckman Coulter Genomics, Fullerton, CA, USA) following the manufacturer’s protocol.

In a second PCR step, dual indices and Illumina sequencing adapters were attached using 7.5 μl of amplicon PCR product DNA, 2.5 μl of Illumina Nextera XT Index Primer 1 (N7xx), 2.5 μl of Nextera XT Index Primer 2 (S5xx), and 12.5 μl of NEBNext High-Fidelity 2X PCR Master Mix, with thermocycling at 95 °C for 3 min, followed by 13 cycles of 95 °C for 30 s, 55 °C for 30 s, and 72 °C for 30 s, and a final extension at 72 °C for 5 min. The final PCR products were cleaned with Agencourt AMPure XP beads, and final concentrations of samples were measured with Qubit™ dsDNA HS Assay Kit.

### Pooling of PCR amplicons

Amplified libraries were pooled at 8 ng/μl, and 30 ng/μl concentrations for low-concentration and high-concentration samples, respectively. After measuring the concentrations of these two pools, they were mixed to 8.1 ng/μl for each amplicon with maximum volume from negative controls. The final pool was purified with Agencourt AMPure XP beads, and eluted in 30 μl to increase the final concentration.

### Sequencing

Concentration and quality of the pooled PCR amplicons was evaluated with Qubit™ dsDNA HS Assay Kit, and a fragment analyser (High Sensitivity NGS Fragment Analysis Kit, Advanced Analytical Technologies, Inc., Ankeny, IA, USA). The fragment analyser traces indicated that no primer-dimers or other non-target PCR products were present; thus, size selection was not required. A quantitative real-time PCR (qPCR) assay, designed to specifically detect adapter sequences flanking the Illumina libraries, was performed using an Illumina® KAPA Library Quantification Kit (Kapa Biosystems, Wilmington, USA). This assay was used to quantify the number of DNA templates that had both adaptor sequences on either end and therefore those that would successfully form clusters on a flowcell for sequencing. Each pool of amplicon libraries was sequenced on a lane of the Illumina® HiSeq2500 platform, in rapid mode with version 2 chemistry using sequencing by synthesis (SBS) technology to generate 2 × 300 bp paired-end reads. 15% PhiX fragment library was added to increase sample diversity.

### Bioinformatics

Initial processing and quality assessment of the sequence data used an in-house pipeline. CASAVA version 1.8.2 (Illumina) was used for base-calling and de-multiplexing of indexed reads to produce 102 samples across the two runs, in FASTQ format. PCR primer sequences and Illumina adapter sequences were trimmed by using Cutadapt version 1.2.1^56^. Sequencing errors were corrected to improve base quality in both forward and reverse reads using the error-corrected module within SPAdes sequence assembler (version 3.1.0)^57^. Read pairs were converted to single sequences that span entire amplicons using PEAR (version 0.9.10)^58^. Sequences with uncalled bases were removed. Size selection was applied to select sequences between 200 bp and 750 bp, thus removing sequences from potential PCR primer dimers or spurious amplification events. Sequences matching PhiX (E-value < 10^−5^) were filtered out of the dataset.

For each sample, sequences that passed the filters were merged into a single file. This final sequence file with its own metadata file containing description for each sample, was analysed using a custom pipeline based on QIIME 1.9.0^59^. The Silva database (version 123)^60^ was used along all the analysis, except for the phylogenetic tree alignment step which was performed using the GreenGenes precomputed 16S rRNA gene tree. The obtained amplicon sequences were sorted in groups to identify the sequence variability in each sample. This step has been performed using SWARM^61^ with the strictest parameters (default parameters). Potential chimeric-sequences were discarded. Sequences were then aligned on the identified clusters to calculate the abundance of each cluster using a minimum similarity threshold of 97% for the entire length of the sequence in VSEARCH1.1.3. Taxonomic assignment of each cluster was carried out using QIIME and the RDP classifier^62^ to match a representative sequence from each OTU to a sequence from the database. The most abundant sequence within each OTU’s cluster was used as the representative sequence.

### Statistical analyses

To visualize the community composition of each sample, the OTUs abundance table obtained after normalization (at 260,000 sequences per sample) was used to summarise taxon abundance for a given taxonomic rank (from kingdom to species), using QIIME. The OTUs abundance table was also used to investigate the richness and evenness of the samples using the following estimators: total observed sequence variants (*i*.*e*. number of OTUs in the sample), Chao1, Shannon, and Simpson indexes. Comparisons of these indexes between the different groups of samples were performed using a series of t-tests.

To consider how the taxa composition changed in relation to groups for each metadata category, the rarefied abundance table was used to build pairwise sample distance matrices, using the Bray-Curtis^63^ and the Weighted and Unweighted UniFrac^64^ dissimilarity measures. Nonmetric Multidimensional Scaling (NMDS) analysis was performed on the obtained dissimilarity matrices and statistical significance for the obtained dissimilarity matrices calculated using analysis of similarities (ANOSIM). Subsequent *P*-values were calculated using Student’s t-test.

Mean relative abundances of the fifteen most prevalent phyla and genera were charted for each lesion. Log fold changes (Log10) in relative abundance of the genera, with at least 1% abundance in one sample, were calculated for each lesion compared to their respective healthy skin control samples. Robust response screening analysis was performed in JMP Pro 12 (SAS Institute Inc., Cary, NC) in order to evaluate the differences in OTU (genus level assignments) relative abundance between complicated CHDLs, IH, and IP and their corresponding healthy skin control samples. A false discovery rate (FDR) correction was applied and statistical significance was declared at FDR LogWorth of 1.3 (equivalent of a *P*-value of 0.05). To facilitate data presentation, the genera with a Robust FDR LogWorth of 20 or more were adjusted to 20 (corrected Robust FDR LogWorth). Subsequently, the log fold change was plotted versus the corrected Robust FDR LogWorth value using bubble plot graphs in JMP Pro 12. Genera mean relative abundance defined the bubbles’ size, and effect size was indicated by the bubbles’ colouring^28^.

## Electronic supplementary material

Supplementary Information

## Data Availability

The datasets are available on the European Nucleotide Archive under the study accession number: PRJEB24706 (http://www.ebi.ac.uk/ena/data/view/PRJEB24706).
