# Supplementary material for: 16S rRNA amplicon sequencing reveals a polymicrobial nature of complicated claw horn disruption lesions and interdigital phlegmon in dairy cattle
Source: Sci Rep. 2018 Oct 19;8:15529. doi: 10.1038/s41598-018-33993-9 (PMC6195575; doi:10.1038/s41598-018-33993-9)

16S rRNA amplicon sequencing reveals a polymicrobial nature of complicated claw horn disruption lesions and interdigital phlegmon in dairy cattle

Bay V.[1]*, Griffiths B.[2]*, Carter S.[3], Evans N.J.[3], Lenzi L.[4], Bicalho R. C.[5], Oikonomou G.[1,2]
[1]Department of Epidemiology and Population Health, Institute of Infection and Global Health, University of Liverpool
[2]Department of Livestock Health and Welfare, Institute of Veterinary Sciences, University of Liverpool
[3]Department of Infection Biology, Institute of Infection and Global Health, University of Liverpool.
[4]Centre for Genomic Research, Institute of Integrative Biology, University of Liverpool
[5]Department of Population Medicine and Diagnostic Sciences, College of Veterinary Medicine, Cornell University
*These authors contributed equally to this work

Corresponding author: goikon@liverpool.ac.uk

Supplementary Figure 1. Relative abundances of the fifteen most prevalent bacterial genera in IP lesions and their healthy skin control samples. (IP: Interdigital Phlegmon, IPC: IP Control)

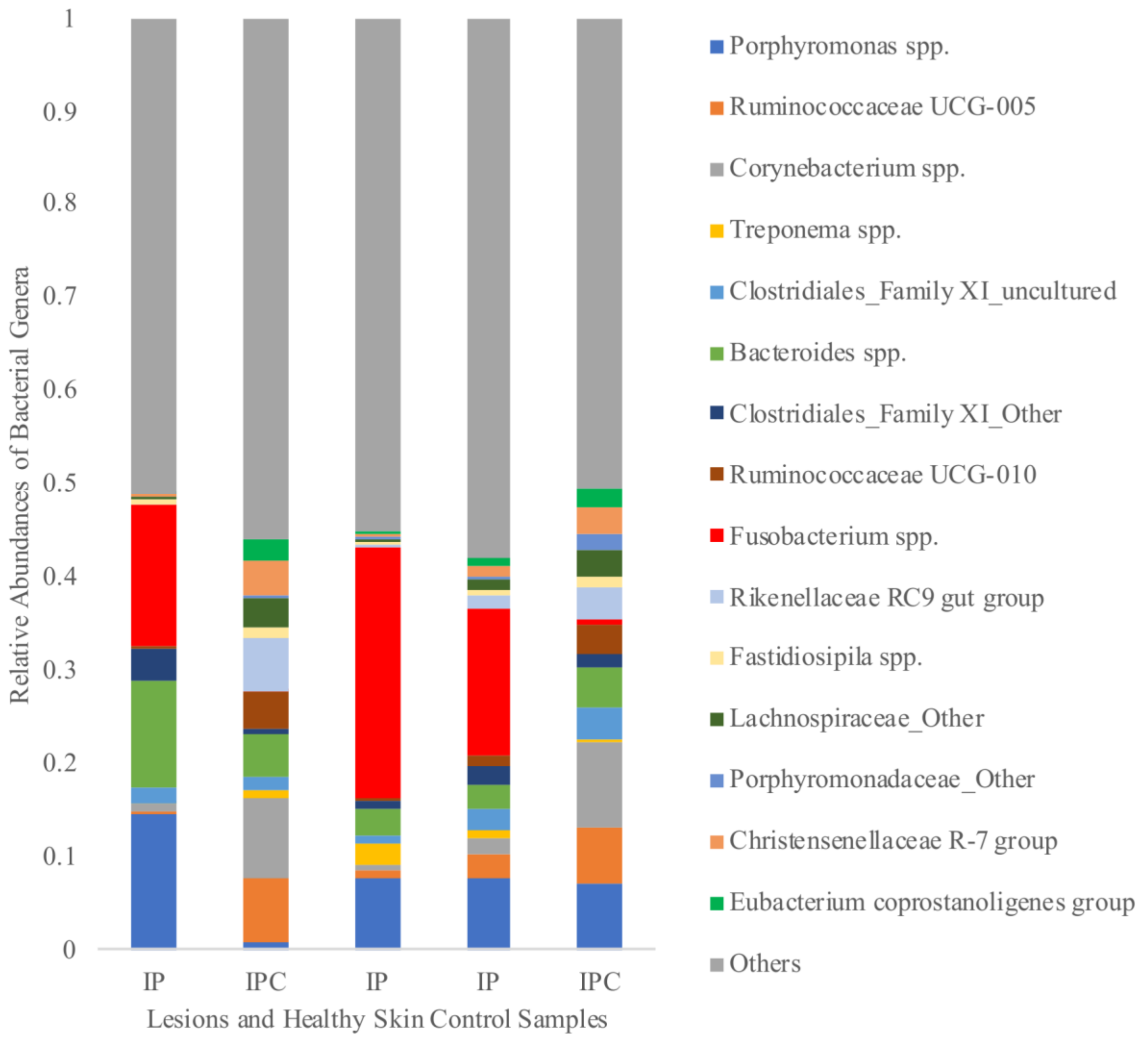

Supplementary Figure 2. Relative abundances of the fifteen most prevalent bacterial genera in SU lesions and their healthy skin control samples. (SU: Sole Ulcer, SUC: SU Control)

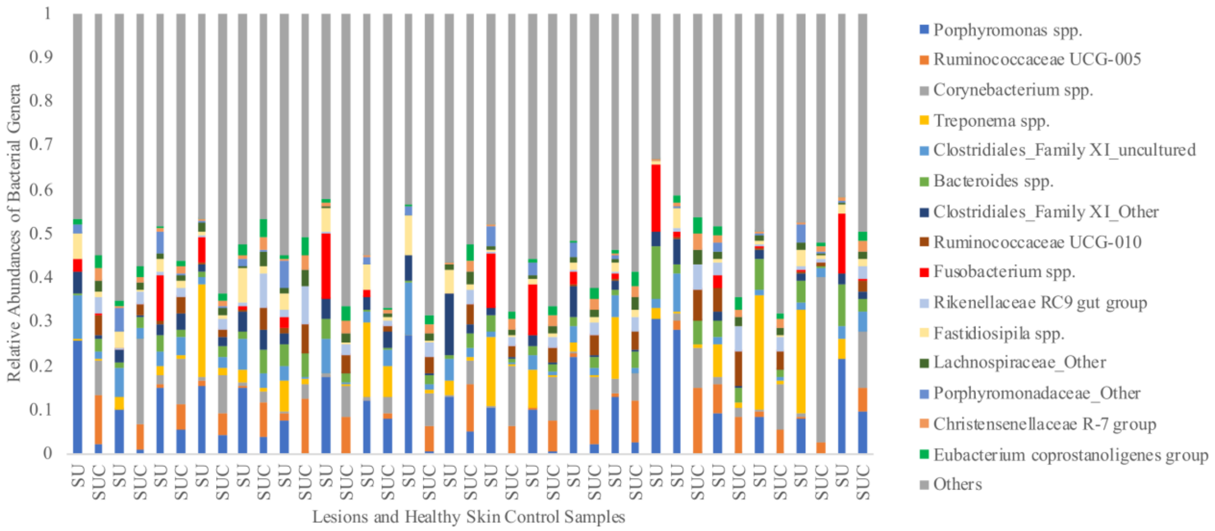

Supplementary Figure 3. Relative abundances of the fifteen most prevalent bacterial genera in TN lesions and their healthy skin control samples. (TN: Toe Necrosis, TNC: TN Control

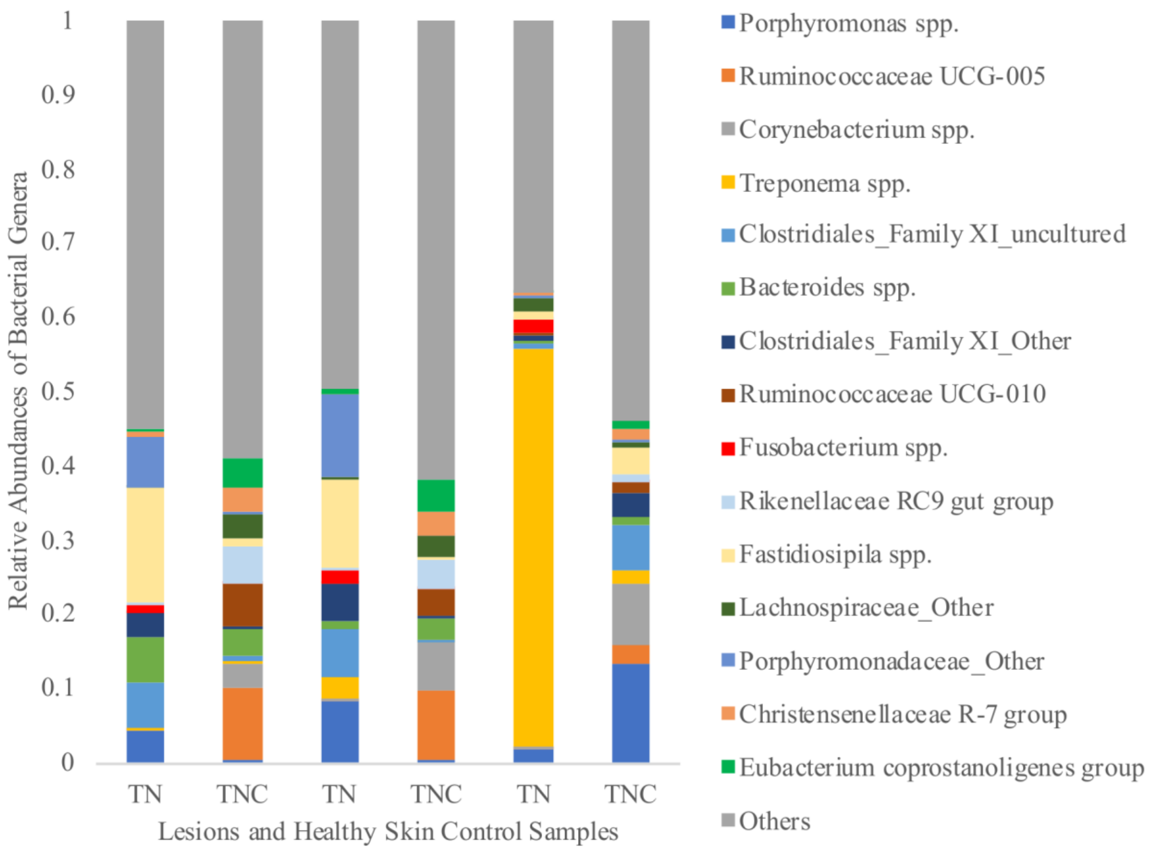

Supplementary Figure 4. Relative abundances of the fifteen most prevalent bacterial genera in WLD lesions and their healthy skin control samples. (WLD: White Line Disease, WLDC: WLD Control)

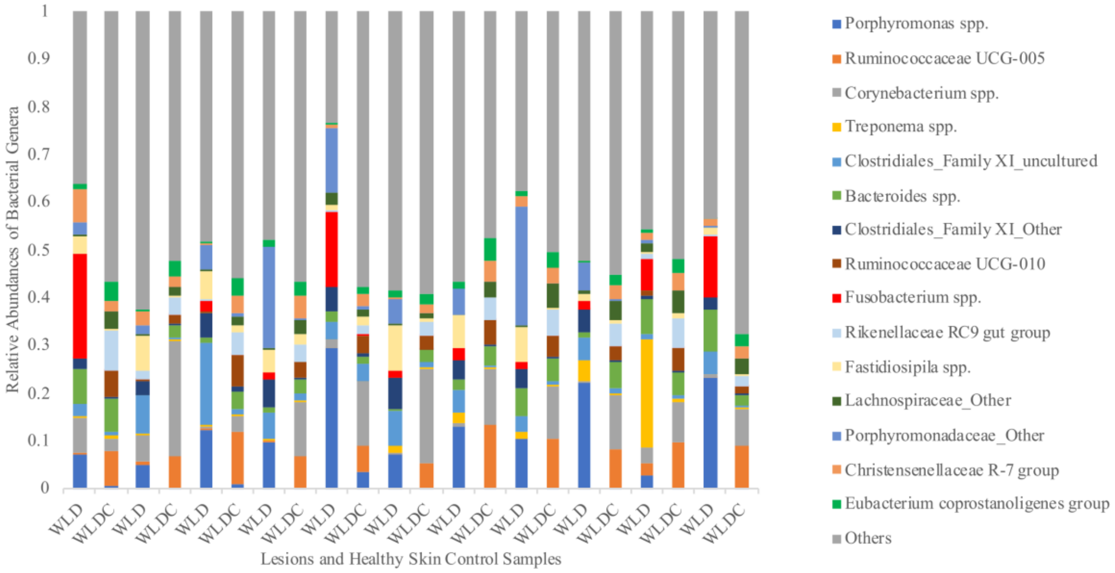

Supplement: Supplementary file 1 — Supplementary Information [file 41598_2018_33993_MOESM1_ESM.pdf]
